# Methionine abrogates the renoprotective effect of a low-protein diet against diabetic kidney disease in obese rats with type 2 diabetes

**DOI:** 10.18632/aging.102902

**Published:** 2020-03-06

**Authors:** Munehiro Kitada, Yoshio Ogura, Itaru Monno, Jing Xu, Daisuke Koya

**Affiliations:** 1 Department of Diabetology and Endocrinology, Kanazawa Medical University, Uchinada, Ishikawa, Japan; 2 Division of Anticipatory Molecular Food Science and Technology, Medical Research Institute, Kanazawa Medical University, Uchinada, Ishikawa, Japan

**Keywords:** diabetic kidney disease, S-adenosylmethionine, glycine N-methyltransferase, mechanistic target of rapamycin complex 1, autophagy

## Abstract

Dietary interventions, including a low-protein diet (LPD) and methionine (Met) restriction, have shown longevity, anti-aging and metabolic health effects. We previously reported that the LPD has a renoprotective effect against diabetic kidney disease (DKD) in rats with type 2 diabetes and obesity. However, it is unclear whether the beneficial effect of the LPD is mediated by low-Met intake or how Met is related to the pathogenesis for DKD. We herein show that the addition of Met with the LPD abrogates the beneficial effects induced by the LPD such as anti-oxidative stress, anti-inflammation and anti-fibrosis, in diabetic kidney. Additionally, the increased levels of S-adenosylmethionine (SAM) in renal tubular cells, which are associated with the reduced expression of glycine N-methyltransferase (Gnmt) and non-restricted Met intake, contributes to the activation of mechanistic target of rapamycin complex 1 (mTORC1) and impaired autophagy, in diabetic kidney. Moreover, starvation-induced autophagy was suppressed in renal cortex of Gnmt null mice and amino acid free-induced autophagy was also suppressed by administration of SAM in cultured HK-2 cells. A LPD could exert a renoprotective effect through the suppression of mTORC1 and restoration of autophagy, which is associated with reduced levels of SAM due to low-Met intake, in diabetic kidney.

## INTRODUCTION

Diabetic kidney disease (DKD) is recognized as the leading cause of end-stage renal disease (ESRD). The degree of tubulointerstitial damage rather than glomerular damage is predictive of a decline in renal function in patients with chronic kidney disease (CKD), including DKD [1, 2]. Therefore, protecting renal tubular cells against cellular stress preserves renal function. Because renal tubular cells are rich in mitochondria due to the high energy demand for the reabsorption of many materials from filtered urine, impaired mitochondrial function in renal tubular cells contributes to renal injury. In the pathogenesis of DKD, renal tubular damage and tubulointerstitial fibrosis play a crucial role in glomerular damage, which is called “diabetic tubulopathy” [3]. Additionally, aging is a universal process that affects all organs, and a gradual age-related decline in renal function, which is accompanied by an increase in histological renal tubulointerstitial and glomerular fibrosis, is observed [4, 5]. Thus, since a common pathway in the progression of the aging kidney and DKD exists, aging may be a risk factor for the development of ESRD due to DKD. Therefore, the regulation of the aging process in the kidney may be a candidate therapeutic intervention against DKD.

Dietary interventions have been recognized as one of the experimental methods for lifespan extension and the suppression of age-related diseases. Numerous reports have shown that calorie restriction (CR) or dietary restriction (DR) without malnutrition extends the lifespan and improves metabolic health in organisms [6]. However, recent evidence indicates that protein restriction or a low-protein diet (LPD) is more strongly associated with the benefits of lifespan extension and metabolic health than CR [7, 8]. Moreover, restriction of specific amino acids, particularly methionine (MetR), has been demonstrated to extend the lifespan [9–12] and promote the metabolic health of organisms through multiple mechanisms related to anti-aging effects, including improvement in oxidative stress/inflammation [13–15], improvement in glucose metabolism [12, 16–20], suppression of mechanistic target of rapamycin complex 1 (mTORC1) [21, 22] or induction of autophagy [21, 22], which may lead to the protection of organs, including the kidney [23, 24]. We also previously showed that a LPD improved advanced diabetes-induced renal injuries, including the accumulation of abnormal mitochondria, tubular cell damage and inflammation, which is associated with autophagy restoration and suppression of the mTORC1 pathway, in a rat model of type 2 diabetes (T2DM) and obesity [25]. However, it remains unclear whether the beneficial effect of a LPD against DKD is exerted through MetR. In this study, we demonstrated that the addition of Met to the LPD at the same level as the standard diet (STD) clearly abrogated the renoprotective effect of the LPD on diabetes-induced renal injuries, including inflammation, mitochondrial abnormality and fibrosis. Additionally, we found that the accumulation of S-adenosylmethionine (SAM), a Met metabolite associated with a decrease in glycine-N-methyltransferase (Gnmt) expression/activation in renal tubular cells, possibly contributes to the activation of mTORC1 and impairment in autophagy and leads to the pathogenesis of DKD in rats with T2DM and obesity.

## RESULTS

### Effect of addition of Met to a LPD on characteristics and biological parameters in diabetic rats

**Figure 1:**
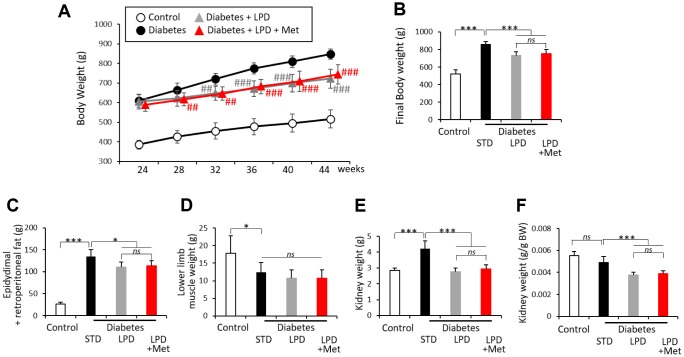
**Characteristics of rats.** Whole body weights of each group of rats during the 20-week experimental period (**A**). Whole body weights (**B**), epidydimal and post peritoneal fat weights (**C**), lower limb muscle weights (**D**), kidney weights (**E**) and kidney weights per g body weight (BW) (**F**) at the end of the study (n=7). The data shown are the means ± SD. ^##^p<0.01, ^###^ p<0.001 vs. diabetes, *p<0.05, **p<0.01, ***p<0.001 vs. the indicated groups. *ns*: not significant. White circles: control, black circles: diabetes, white triangles: diabetes + LPD, black triangles: diabetes + LPD + Met. LPD: low-protein diet, Met: methionine, BW: body weight.

The overall body weight (BW) changes of rats are presented in Figure 1A. At the start of the experimental period, overall BW was not significantly different among all three groups of Wistar fatty (diabetic) rats. The diabetes + STD group gained more BW than did the diabetes + LPD and diabetes + LPD + Met groups during the experimental period (Figure 1A). The overall BW and epididymis/retroperitoneal fat weights of the diabetes + LPD and diabetes + LPD + Met groups were significantly lower than those in the diabetes + STD group at the end of the study (Figure 1B and 1C). There was no difference on overall BW and epididymis/retroperitoneal fat weights between the LPD and LP + Met diets in diabetic rats (Figure 1B and 1C). Neither the LPD nor the LP + Met diet affected lower limb muscle weights in diabetic rats at the end of the study (Figure 1D). The kidney weight of the diabetes + LPD and diabetes + LPD + Met groups was significantly lower than that in the diabetes + STD group at the end of the study (Figure 1E and 1F). There was no difference on kidney weight between the LPD and LP + Met diets in diabetic rats (Figure 1E and 1F). The overall BW, epididymis/retroperitoneal fat weights and kidney weight of diabetes + STD group were significantly higher than those in the Wistar lean (control) group, and the lower limb muscle weights of diabetes + STD group were significantly lower than those of control group (Figure 1B–1E). However, there was no difference of kidney weight per g BW between control group and diabetes + STD group (Figure 1F).

**Figure 2:**
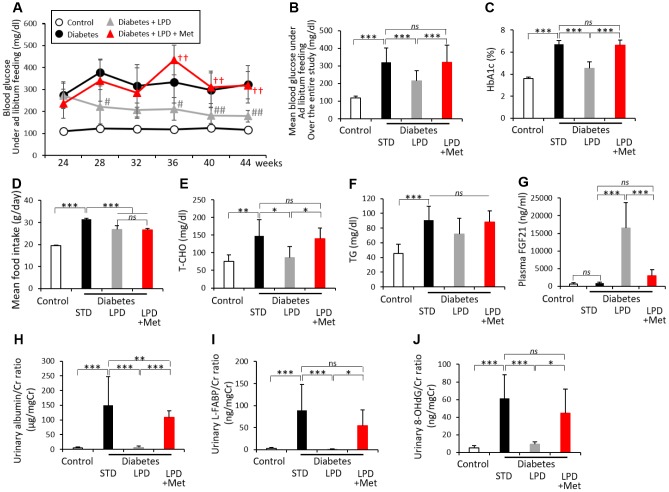
**Biological parameters of rats.** Blood glucose levels under ad libitum feeding during the 20-week experimental period (**A**), mean blood glucose levels under ad libitum feeding over the entire study (**B**), HbA1c levels (**C**), mean food intake over the entire study (**D**), fasting T-CHO (**E**), TG (**F**), postprandial plasma FGF21 levels (**G**), urinary albumin/Cr ratio (**H**), urinary L-FABP/Cr ratio (**I**) and urinary 8-OHdG/Cr ratio (**J**), at the end of the study (n=7). The data shown are the means ± SD. ^#^p<0.05, ^##^p<0.01 vs. diabetes, ^††^p<0.01 vs. diabetes + LPD, *p<0.05, **p<0.01, ***p<0.001 vs. the indicated groups. *ns*: not significant. White circles: control, black circles: diabetes, white triangles: diabetes + LPD, black triangles: diabetes + LPD + Met. T-CHO: total cholesterol, TG: triglyceride, FGF21: fibroblast growth factor 21, Cr: creatinine, L-FABP: liver-type fatty acid binding protein, 8-OHdG: 8-hydroxy-2'- deoxyguanosine.

The diabetes + LPD group exhibited lower blood glucose levels under ad libitum feeding than those of the diabetes + STD group. However, the diabetes + LPD + Met group showed high levels of blood glucose under ad libitum feeding, which were similar to those of the diabetes + STD group (Figure 2A). The LPD resulted in an improvement in mean blood glucose levels under ad libitum feeding during the experimental period and hemoglobin A1c (HbA1c) levels at the end of the study, in diabetic rats (Figure 2B and 2C). However, the LP + Met diet attenuated the effect of the LPD on diabetic status, showing an elevation of mean blood glucose levels under ad libitum feeding and HbA1c in diabetic rats (Figure 2B and 2C). The mean food intake (g/day) was reduced in both the diabetes + LPD and diabetes + LPD + Met groups compared to that in the diabetes + STD group during the experimental period (Figure 2D). However, the mean food intake between the diabetes + LPD and diabetes + LPD + Met rats was not different (Figure 2D). The mean food intake (g/g BW/day) in the diabetes + STD, diabetes + LPD and diabetes + LPD + Met groups was 0.042 ± 0.004, 0.040 ± 0.003 and 0.040 ± 0.002 (g/g BW/day) and was not statistically significant. Plasma fasting total cholesterol (T-CHO) and triacylglycerol (TG) levels were also significantly elevated in diabetes + STD compared to levels in control rats (Figure 2E and 2F) at the end of the study. The increases in T-CHO levels in the diabetes + STD group were reduced by the LPD. However, the diabetes + LPD + Met treatment produced elevated T-CHO levels. The LPD and LP + Met diet did not lead to a statistically significant difference in the TG levels of diabetic rats (Figure 2E and 2F). The plasma fibroblast growth factor21 (FGF21) levels were markedly increased in the diabetes + LPD group; however, the LP + Met diet decreased the levels of plasma FGF21 in diabetic rats at the end of the study (Figure 2G). There was no statistically significant difference in the levels of plasma FGF21 between diabetes + STD and diabetes + LPD + Met groups. The mean blood glucose levels under ad libitum feeding during the experimental period, HbA1c, plasma fasting T-CHO and TG levels at the end of the study were significantly higher in the diabetes + STD group than the levels in the control group (Figure 2A–2F).

The levels of urinary albumin and liver-type fatty acid binding protein (L-FABP) excretion in diabetes + STD rats were markedly increased compared to those of the control rats at the end of the study (Figure 2H and 2I). LPD clearly reduced the urinary albumin and L-FABP excretion levels in diabetic rats; however, the LP + Met diet abrogated the effects on urinary albumin and L-FABP excretion in diabetic rats, thereby showing an elevation of urinary albumin and L-FABP excretion (Figure 2H and 2I). In addition, urinary 8-hydroxy-2’-deoxyguanosine (8-OHdG) excretion was significantly enhanced in the diabetes + STD group compared to that in the control at the end of the study (Figure 2J). The LPD reduced the increased levels of urinary 8-OHdG in diabetic rats; however, the diabetes + LPD + Met rats again exhibited an exacerbation of urinary 8-OHdG excretion (Figure 2J).

### Changes in renal fibrosis and tubular cell damage

**Figure 3:**
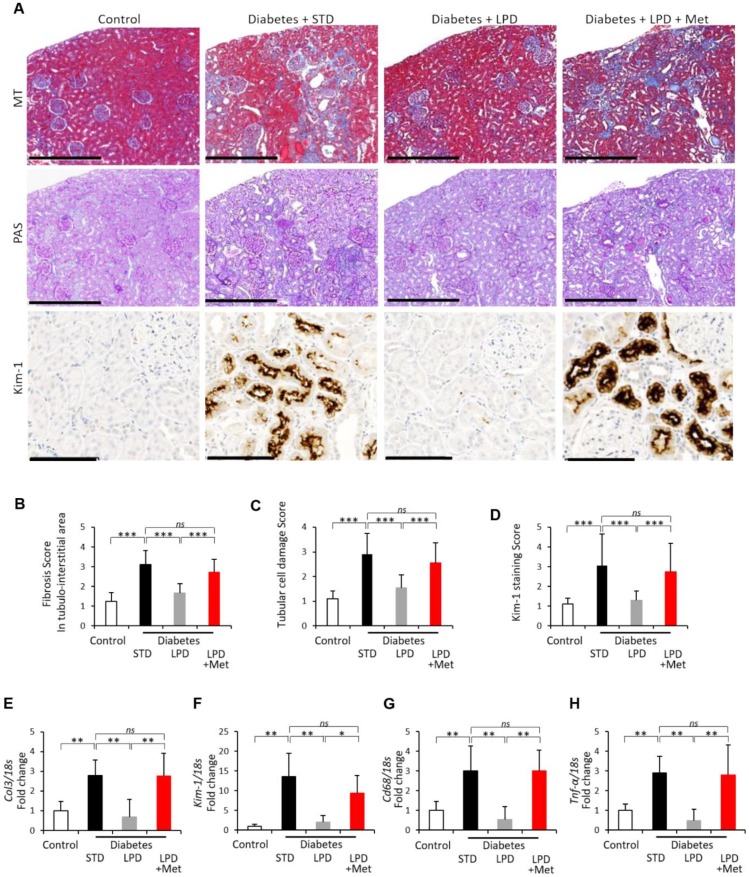
**Changes in renal fibrosis and tubular cell damage.** Representative photographs of MT, PAS staining and Kim-1 immunohistochemistry of the kidney after the intervention. MT staining of the tubulointerstitial area (scale bar: 500 μm), PAS staining for the evaluation of tubular cell damage (scale bar: 500 μm) and immunohistochemistry for Kim-1 (scale bar: 100 μm) at the end of the study (**A**); Tubulointerstitial fibrotic scores obtained using MT staining (**B**) (n=7); tubular cell damage scores obtained using PAS staining (**C**) (n=7); semiquantitation of Kim-1 staining scores (**D**) (n=3); mRNA expression of *Col3* (**E**), *Kim-1* (**F**), *Cd68* (**G**) and *Tnf-α* (**H**) adjusted to *18S* levels, in the renal cortex at the end of the study (n=7). The data shown are the means ± SD. *p<0.05, **p<0.01, ***p<0.001 vs. the indicated groups. *ns*: not significant. MT: Masson’s trichrome, PAS: Periodic Acid Schiff, Col3: type 3 collagen, Kim-1: kidney injury molecule-1, Tnf-α: tumor necrosis factor-α.

Representative photomicrographs of Masson’s trichrome (MT) staining, Periodic Acid Schiff (PAS) staining and kidney injury molecule-1 (Kim-1) immunohistochemically stained kidney sections from each rat group are shown in Figure 3A. The renal fibrosis scores in the tubule-interstitial area and renal tubular damage scores evaluated by MT and PAS staining were significantly higher in the diabetes + STD group than those in the control group (Figure 3B and 3C). The Kim-1 staining scores in the diabetes + STD group were also significantly higher than the scores in the control (Figure 3D). Diabetes + STD rats showed that the mRNA expression of type 3 collagen (*Col3*), *Kim-1*, *Cd68* and tumor necrosis factor-α (*Tnf-α*) in the renal cortex was significantly increased compared to that in the control (Figure 3E to 3H). Renal fibrosis, tubular cell damage and inflammation in diabetic rats were improved by the LPD; however, the LP + Met diet exacerbated all renal alterations (Figure 3A to 3H).

### Changes in glycine N-methyltransferase (Gnmt) and S-adenosylmethionine (SAM) expression in the kidney

**Figure 4:**
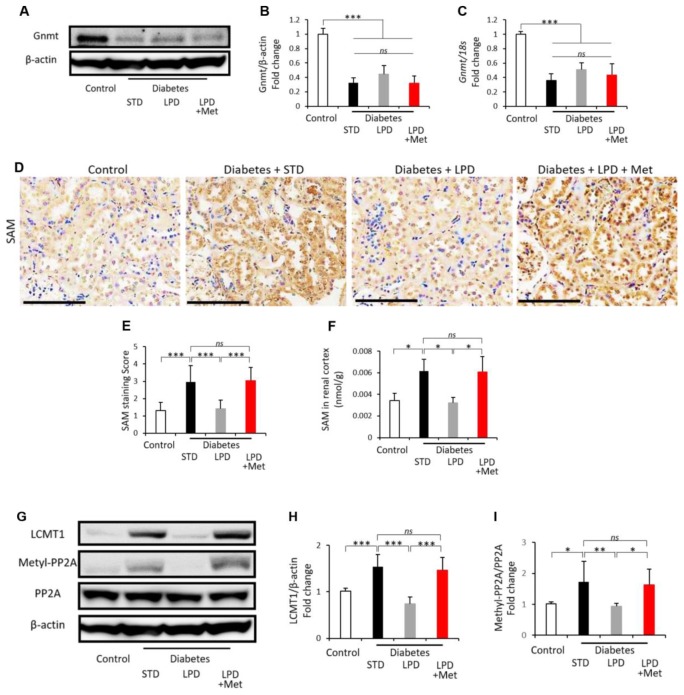
**Changes in the renal Gnmt expression and SAM content.** Representative western blots of Gnmt and β-actin in the renal cortex (**A**). Quantitative ratios of Gnmt to β-actin (**B**) and mRNA expression of Gnmt adjusted to 18S levels (**C**) in the renal cortex (n=7). Representative photographs of SAM immunohistochemistry of the kidney (scale bar: 100 μm) (**D**). Semiquantitation of the SAM staining scores (**E**) (n=3). SAM levels in the renal cortex (**F**) (n=3). Representative western blots of LCMT1, methylated-PP2A, PP2A and β-actin in the renal cortex (**G**). Quantitative ratios of LCMT1 to β-actin (**H**) and methylated-PP2A to PP2A (**I**) in the renal cortex (n=7). The data shown are the means ± SD. **p<0.01, ***p<0.001 vs. the indicated groups. *ns*: not significant. Gnmt: glycine-N-methyltransferase, SAM: S-adenosylmethionine, LCMT1: leucine carboxyl methyltransferase 1, PP2A: protein phosphatase 2A.

Gnmt expression in the renal cortex was evaluated by western blotting and real time-polymerase chain reaction (PCR) (Figure 4A–4C). Both the protein and mRNA levels of Gnmt expression were markedly reduced in the diabetic renal cortex compared to those of the control (Figure 4A–4C). Neither the LPD nor the LP + Met diet affected Gnmt expression levels in diabetic rats (Figure 4A–4C). The immunohistochemical staining intensity of SAM was clearly enhanced in renal tubular cells in diabetes + STD rats compared to that in controls (Figure 4D and Figure 4E). SAM levels in renal cortex was also elevated in diabetes +STZ rats compared to those in controls (Figure 4F). The LPD reduced the SAM staining intensity and SAM levels; however, the LP + Met diet increased the levels of SAM staining intensity and SAM levels in diabetic rats (Figure 4D-4F). Leucine carboxyl methyltransferase 1 (LCMT1) can methylate the catalytic subunit of protein phosphatase 2A (PP2A) in response to SAM, leading to activation of PP2A (Figure 4E–4G-4I). In the renal cortex of diabetes + STD rats, both the expression levels of LCMT1 and methyl-PP2A were significantly increased compared to those of the control (Figure 4E–4G-4I). The diabetes + LPD rats exhibited a decrease in the expression of LCMT1 and methyl-PP2A; however, the diabetes + LPD + Met rats showed an elevation in LCMT1 and methyl-PP2A expression (Figure 4E–4G-4I).

### Changes in mTORC1 activity in the kidney

**Figure 5:**
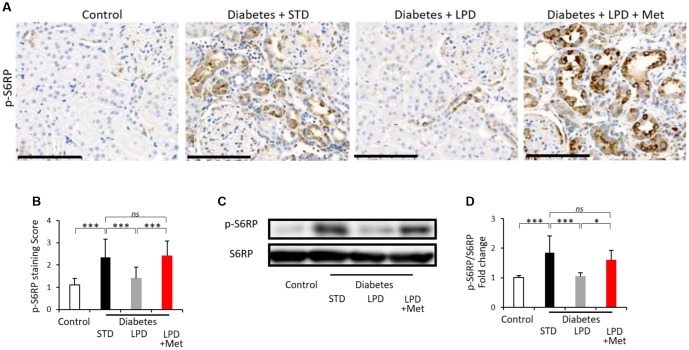
**Changes in mTORC1 activity in the kidney.** Representative photographs of immunohistochemistry for p-S6RP in tubular lesions (**A**) (scale bar: 100 μm). Semiquantitation of p-S6RP staining scores in tubulointerstitial lesions (**B**) (n=3). Representative western blots of p-S6RP and S6RP in the renal cortex (**C**). Quantitative ratios of p-S6RP to S6RP (**D**) (n=7). The data shown are the means ± SD. *p<0.05, ***p<0.001 vs. the indicated groups. *ns*: not significant. mTORC1: mechanistic target of rapamycin complex 1, S6RP: phospho-S6 ribosomal protein.

Diabetes + STD showed an increased intensity of immunohistochemical staining for phospho-S6 ribosomal protein (p-S6RP) (a downstream effector molecule of mTORC1) in tubular lesions compared to that in the control rats, and LPD decreased the intensity of its staining to levels similar to those observed in the controls (Figure 5A and 5B). However, LPD + Met increased the staining levels of p-S6RP in diabetic kidneys (Figure 5A and 5B). The p-S6RP expression evaluated by western blotting was also enhanced in the renal cortex of the diabetes + STD group compared to expression in the control group, and the increased p-S6RP expression in diabetic renal cortex was significantly decreased by the LPD (Figure 5C and 5D). The LP + Met diet induced increased p-S6RP expression in the diabetic renal cortex (Figure 5C and 5D).

### Alterations in mitochondrial morphology in proximal tubular cells (PTCs) and autophagy in the kidney

**Figure 6:**
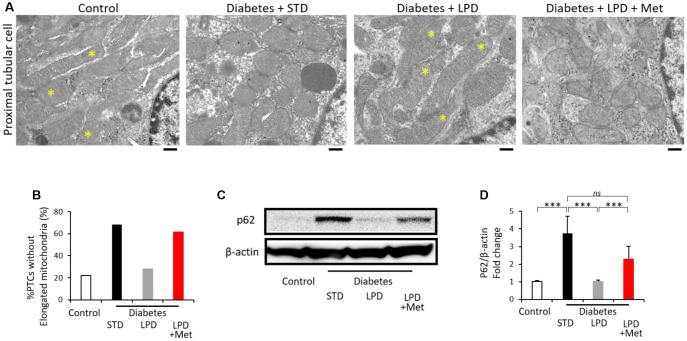
**Morphology of mitochondria in PTCs and autophagy.** Representative TEM images of PTCs (scale bar: 500 nm) (**A**). Asterisks indicate elongated (>2 μm) mitochondria (**A**). The ratio of PTCs without elongation of mitochondria to total PTCs (**B**) (from 3 animals). Representative western blots of p62 and β-actin expression (**C**) and the quantitative ratios of p62 to β-actin in the renal cortex (**D**) (n=7). The data shown are the means ± SD. **p<0.01, ***p<0.001 vs. the indicated groups. *ns*: not significant. PTCs: proximal tubular cells, TEM: transmission electron microscopy.

Diabetes + STD exhibited alterations in mitochondrial morphology in PTCs, such as fragmented mitochondria and increased numbers of PTCs without elongated mitochondria (Figure 6A and 6B). The LPD improved the alterations in mitochondrial morphology (Figure 6A and 6B); however, the LP + Met diet in diabetic rats abrogated the effect of the LPD on the alteration of mitochondrial abnormality (Figure 6A and 6B). Immunohistochemical staining for p62 was significantly enhanced in the renal cortex of the diabetes + STD group, indicating the impairment of autophagy. The LPD ameliorated p62 expression in the kidneys of diabetic rats (Figure 6C and 6D). However, diabetes + LPD + Met rats exhibited increased p62 expression in the renal cortex (Figure 6C and 6D).

### Effect of SAM on amino acid-free-induced autophagy and methyl-PP2A and p-S6RP expression in cultured HK-2 cells, and change of starvation-induced autophagy in the renal cortex of Gnmt knockout (KO) mice

**Figure 7:**
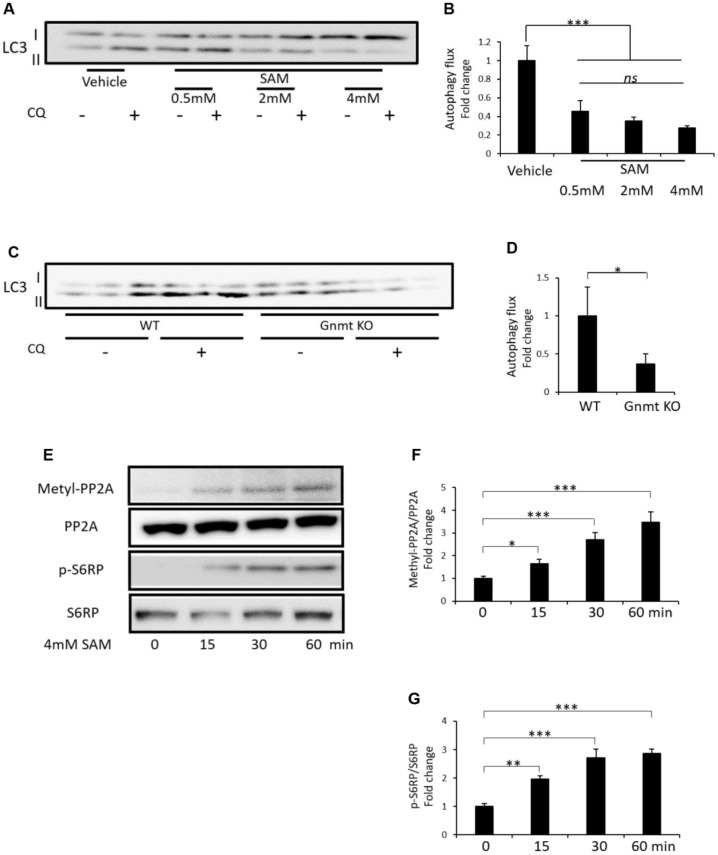
**Effect of SAM on amino acid-free-induced autophagy and methyl-PP2A and p-S6RP expression in cultured HK-2 cells and change of starvation-induced autophagy in the renal cortex of Gnmt knockout (KO) mice.** Representative western blots of LC3 in HK-2 cells (**A**). Quantitative autophagic flux in cultured HK-2 cells (**B**) (n=3). Representative western blots of LC3 in the renal cortex of Gnmt KO mice after 48 hours of starvation (**C**). Quantitative autophagic flux in the renal cortex of mice (**D**) (n=3). Representative western blots of methyl-PP2A, PP2A, p-S6RP and S6RP in HK-2 cells (**E**). Quantitative expression of methyl-PP2A to PP2A (**F**) and p-S6RP to S6RP (**G**) in HK-2 cells (n=3). The data shown are the means ± SD. *p<0.05, ***p<0.001 vs. the indicated groups. *ns*: not significant. SAM: S-adenosylmethionine, Gnmt: glycine-N-methyltransferase, CQ: chloroquine, LC3: Microtubule-associated protein 1 light chain 3, WT: wild type, Gnmt: glycine N-methyltransferase, SAM: S-adenosylmethionine, Methyl-PP2A: methylated protein phosphatase 2A, PP2A: protein phosphatase 2A, p-S6RP: phospho-S6 ribosomal protein, S6RP: S6 ribosomal protein.

To clarify whether SAM itself suppresses autophagy, we analyzed microtubule associated protein light chain 3-II (LC3-II) turnover through preventing lysosomal degradation by using chloroquine (CQ), in cultured HK-2 cells (human kidney proximal tubular cells) and renal cortex of Gnmt KO mice. Administration of SAM suppressed amino acid-free-induced autophagy in cultured HK-2 cells (Figure 7A and 7B). Additionally, in the renal cortex of Gnmt KO mice, which accumulate SAM in the organs, including the liver and kidney, 48-hour starvation-induced autophagy in the renal cortex was suppressed compared to wild type mice (Figure 7C and 7D). Furthermore, administration of SAM induced the increased expression of methyl-PP2A and p-S6RP, which were normalized to PP2A or S6RP, from 15 min to 60 min in cultured HK-2 cells (Figure 7E–7G).

## DISCUSSION

Recent reports have shown that a LPD could exert beneficial effects on longevity and metabolic health [26]. Previously, we also demonstrated that a LPD prevented the progression of the diabetic state and onset of DKD and that a LPD also improved advanced diabetes-induced renal injury [25, 27]. On the other hand, restriction of specific amino acids, particularly MetR, has the most benefits for metabolic health and longevity, and its effect in an LPD may be mediated through MetR, which has antioxidation, anti-inflammation, suppression of mTORC1, induction of autophagy and improvement in glucose and lipid metabolism effects [26], which are recognized as anti-aging effects. Our present data clearly showed that the addition of Met to the LPD abolished the beneficial effect of the LPD on diabetes-induced inflammation, mTORC1 activation and impairment of autophagy in the kidney. Furthermore, the addition of Met to the LPD abrogated the LPD-induced reduction in glucose levels in diabetic rats. Thus, this is the first report to reveal that the level of Met intake may be involved in the pathogenesis of DKD by possibly modulating the aging-related process.

As the mechanism by which additional Met intake cancelled the benefit of a LPD on DKD, we focused on Met metabolism in the kidney. Met is converted to SAM in a reaction catalyzed by the enzyme Met adenosyl transferase (MAT), and SAM is converted to S-adenosylhomocysteine (SAH) by Gnmt, a key enzyme for SAM metabolism that maintains the intracellular SAM levels [28]. SAM is an important methyl donor in essentially every tissue, and this path of its metabolism is probably quite important, along with dietary levels of sulfur amino acids such as Met and cysteine (Cys), in determining tissue SAM levels, in addition to Gnmt activity. A recent report has shown that SAM, rather than Met, may be the main contributor to MetR-induced lifespan extension. Obata et al. demonstrated that enhancing SAM catabolism by Gnmt activation extends the lifespan of *Drosophila* [29]*.* Compared with young flies, older flies have increased SAM levels, even though Gnmt is transcriptionally induced with aging [29]*.* However, overexpression of Gnmt suppresses the age-dependent SAM increase and extends the lifespan. A previous report showed that Gnmt is distributed in the liver, kidney, pancreas and jejunum of rats, and in the kidney, Gnmt expression was observed primarily in the PTCs [30]. In this study, we found that both protein and mRNA expression levels of Gnmt in the renal cortex of diabetic rats were markedly reduced compared to those of control rats and that there was no difference in the expression levels of Gnmt in diabetic rats under the dietary intervention, such as the STD, LPD or LP + Met diet. Additionally, the accumulation of SAM in renal tubular cells in the renal cortex of STD-fed diabetic rats was higher than that in control rats. LPD significantly decreased renal SAM accumulation in diabetic rats; however, adding Met to a LPD exacerbated it again in diabetic kidneys. The expression levels of renal Gnmt in diabetic rats were low; therefore, the accumulation of SAM in tubular cells may be dependent on the amount of dietary Met intake in diabetic rats. Jiang et al. previously demonstrated that plasma SAM levels were significantly increased in diabetic patients compared to nondiabetic control subjects [31]. Furthermore, patients with diabetic nephropathy exhibited higher levels of plasma SAM than diabetic patients without nephropathy [31]. Moreover, plasma SAM, but not Met, is independently associated with fat mass and truncal adiposity [32], while overfeeding increases serum SAM in proportion to the fat mass gained [33]. Another report demonstrated that plasma SAM levels were associated with cardiometabolic risk factors such as higher fasting insulin levels, homeostasis model assessment of insulin resistance and TNF-α in a cross-sectional study in humans with metabolic syndrome [34]. Thus, an increase in SAM related to overfeeding or increased fat mass may be associated with diabetes, cardiovascular risk and DKD.

Impairment of autophagy is closely related to aging and age-related diseases, including kidney disease [35]. We also previously demonstrated that autophagy is impaired in diabetic kidneys, which is associated with activation of mTORC1, and that a LPD improved renal injury through restoration of autophagy and suppression of mTORC1 [25]. A previous report showed that high intracellular Met or SAM inhibited non-nitrogen starvation-induced autophagy [21]. In cultured HK-2 cells, administration of SAM suppressed amino acid deprivation-induced autophagy, in this study. Additionally, 48-hour starvation-induced autophagy was suppressed in the renal cortex of Gnmt KO mice compared to that of wild type mice. These data may support that the accumulation of SAM in renal tubular cells possibly contributes to the suppression of autophagy in diabetic rats and that a LPD may activate autophagy through low levels of SAM via low Met intake in the kidney. Additionally, methylation of the catalytic subunit of PP2A by Ppm1, or LCMT1 in mammals, is responsive to SAM concentrations [21, 22]. Methyl-PP2A can activate mTORC1 by dephosphorylating Npr2, which is a component of a negative regulator complex of mTORC1, promoting autophagy [21, 22]. Our present data clearly showed that STD-fed and LP + Met diet-fed diabetic rats exhibited increased expression of LCMT1 and methyl-PP2A compared to expression in control and LPD-fed diabetic rats, which was accompanied by an increase in renal SAM levels. Consistent with the alteration of LCMT1 and methyl-PP2A, renal mTORC1 activation was significantly increased in STD-fed and LP + Met-fed diabetic rats, which was evaluated by western blotting and immunohistochemical staining for p-S6RP. Furthermore, increased mitochondrial fragmentation in renal tubular cells of diabetic rats was ameliorated by the LPD. However, the LP + Met diet again exacerbated mitochondrial abnormalities in diabetic rats, which was accompanied by impairment of autophagy, as evaluated by p62 expression. In cultured HK-2 cells, we also confirmed that the administration of SAM induced increased expression of methyl-PP2A and p-S6RP. This is the first report showing that the accumulation of SAM is involved in the pathogenesis of DKD through mTORC1 activation and autophagy dysfunction. On the other hand, it was reported that *Gnmt* mRNA levels were suppressed in patients with advanced nonalcoholic fatty liver disease (NAFLD) [36] and *Gnmt* deficiency and/or hypermethioninemia, and elevated SAM levels could play a role in the pathogenesis of NAFLD by inhibiting lipophagy through PP2A methylation in an mTORC1-independent mechanism [37].

Previous studies have shown that dietary protein restriction may show metabolic benefits, such as reduced body fat gain, improved glucose tolerance, and increased energy expenditure, which are mediated by FGF21 [38–40]. Additionally, MetR is known to mimic the metabolic effects of protein restriction, with FGF21 as a required mechanism [17, 41]. In rodents and humans, MetR improves glucose homeostasis and insulin sensitivity and prevents weight gain [12, 16, 19, 20, 42]; therefore, the amount of Met intake may be related to the impairment of glucose and lipid metabolism, in addition to diabetes-induced renal injury. Our data also showed that a LPD resulted in the improvement in glucose and T-CHO levels in diabetic rats; however, the addition of Met to the LPD attenuated its effects on the diabetic state in LPD-fed diabetic rats, which was possibly associated with changes in plasma FGF21 levels. Plasma FGF21 levels were markedly increased by the LPD; however, the LP + Met diet suppressed it.

In conclusions, in this study, we found that the addition of Met to a LPD abrogated the renoprotective effects on diabetes-induced renal injuries in rats with T2DM and obesity, indicating that a LPD could exert beneficial effects on renoprotection through low Met intake. Met might lead to renal injuries in diabetic rats through the accumulation of SAM, which is related to reduced Gnmt expression, possibly contributing to the modulation of mTORC1 activation and autophagy. Thus, activation of Gnmt may be a therapeutic target for DKD through a decrease in SAM levels. Furthermore, Met exacerbates the diabetic state in LPD-fed diabetic rats, which may be associated with the cancellation of LPD-induced plasma FGF21 levels. However, further study is necessary to elucidate whether a low-Met or MetR diet can suppress diabetic renal injuries as well as a LPD.

In a clinical setting, a LPD is considered the dietary therapy for the suppression of CKD, including DKD. However, an inappropriate LPD has the risk of malnutrition, such as protein energy wasting and sarcopenia, in patients. Therefore, a low-Met diet, such as lowering Met intake to levels characteristic of vegan diets, which have naturally low Met levels [43], or avoiding animal protein such as red meat, which is rich in Met, could represent a nutritional strategy with a lower risk of nutritional issues for preventing and managing patients with T2DM with DKD, in addition to providing many health benefits, including metabolic health and lower cardiovascular risk [44]. However, further study is necessary to elucidate whether a low-Met diet can suppress DKD in humans.

## MATERIALS AND METHODS

### Animals and dietary intervention

Male and female Wistar lean (*fa*/+) rats (WLRs) were provided by the Takeda Pharmaceutical Company Biological Institute (Osaka, Japan) and maintained at Kanazawa Medical University [25]. Male diabetic and obese Wistar fatty (*fa/fa*) rats (WFRs), and age-matched nondiabetic WLRs were used. At 24 weeks of age, the rats were divided into four groups: (1) WLRs fed a STD includes 0.64% Met (control); (2) WFRs fed a STD (0.64% Met) (diabetes + STD); (3) WFRs fed a LPD (0.15% Met) (diabetes + LPD); and (4) WFRs fed a LPD + Met (0.64% Met) (diabetes + LPD + Met). Food for the STD, LPD and LP + Met diet was purchased from CLEA (Japan, Inc., Tokyo, Japan). The STD contained 23.84 kcal% protein, 16.80 kcal% fat, 59.36 kcal% carbohydrates and 3.55 kcal/g energy (CLEA Japan, Inc., Tokyo, Japan), as shown in Table 1. The LPD contained 5.77 kcal% protein, 16.48 kcal% fat, 77.75 kcal% carbohydrates and 3.54 kcal/g energy (CLEA Japan, Inc., Tokyo, Japan). The LP + Met diet contained a total of 0.64% Met by the addition of Met to the LPD. The sources of nutrients and the amino acid contents are shown in Table 1. Dietary intervention was performed for 20 weeks beginning at 24 weeks of age. The rats were maintained in temperature-controlled (23 ± 1°C) rooms on a 12 h:12 h light-dark cycle with free access to water and their assigned chow. BW and blood glucose levels under ad libitum feeding at 10 am were measured every four weeks. Food intake was measured every week and is shown as the mean food intake per day. BW, abdominal fat weights including epididymis and post peritoneal fat, lower limb muscle and kidney weights were measured at the end of the study. After 20 weeks of dietary intervention, individual rats were placed in metabolic cages for urine collection. The rats were anesthetized by inhalation of isoflurane, and the kidneys were subsequently removed after collection of blood samples from the left cardiac ventricle. The samples were stored at −80°C until further use in subsequent experiments. The Research Center for Animal Life Science of Kanazawa Medical University approved all experiments, and all experiments were performed in accordance with the relevant guidelines established by the Institutional Animal Care and Use Committee.

**Table 1:** Source of nutrients.

**Source of diets**	**STD (g)**	**LPD (g)**	**LPD + Met (g)**
**Cornstarch**	35.5	54.1	54.1
**Milk caseins**	24.5	5.85	5.85
**Granulated sugar**	20	20	20
**Corn oil**	6	6	6
**Avicel cellulose**	3	3	3
**Powdered cellulose**	2	2	2
**α-starch**	1	1	1
**Vitamin mix**	1	1	1
**Mineral mix**	7	7	7
**Total**	100g	100g	100g
**Content of amino acids in diets**	**STD (% g)**	**LPD (% g)**	**LPD + Met (% g)**
**Isoleucine**	1.20	0.29	0.29
**Leucine**	2.06	0.46	0.46
**Lysine**	1.74	0.42	0.42
**Methionine**	0.64	0.15	0.64
**Cystine**	0.11	0.03	0.03
**Phenylalanine**	1.10	0.26	0.26
**Tyrosine**	1.23	0.29	0.29
**Threonine**	0.91	0.22	0.22
**Tryptophan**	0.27	0.06	0.06
**Valine**	1.47	0.35	0.35
**Histidine**	0.66	0.16	0.16
**Arginine**	0.81	0.19	0.19
**Alanine**	0.66	0.16	0.16
**Asparagine**	1.54	0.37	0.37
**Glutamine**	4.66	1.11	1.11
**Glycine**	0.39	0.09	0.09
**Proline**	2.45	0.59	0.59
**Serine**	1.13	0.27	0.27

### Biochemical measurements

HbA1c levels were measured using a DCA 2000 Analyzer (Siemens Medical Solutions Diagnostics, Tokyo, Japan) at the end of the experiment [25]. Urinary albumin, L-FABP, and plasma FGF21 were measured using enzyme-linked immunosorbent assay (ELISA) kits (urinary albumin: NEPHRAT II, Exocell, Inc., Philadelphia, PA, USA; plasma FGF21 and L-FABP: R&D Systems, Inc., Minneapolis, USA) [25]. Plasma T-CHO and TGs were measured using a Pureauto S TG-N kit (Sekisui Medical, Tokyo, Japan) and an L-type cholesterol H-test kit (Wako Pure Chemical Industries, Osaka, Japan) [25]. The urinary 8-OHdG concentration was measured by using an ELISA kit (8-OHdG Check, Institute for the Control of Aging, Shizuoka, Japan) [27]. Urinary creatinine (Cr) was measured by enzymatic methods [25].

### Morphological analysis

The kidney sections were stained with MT/PAS reagent [25]. For the semiquantitative evaluation of kidney fibrosis through MT staining in 10 randomly selected tubulointerstitial areas per rat, the percentages of the areas stained for fibrosis were graded as follows: 0, 0 to 5% staining; 1, 5 to 25%; 2, 25 to 50%; 3, 50 to 75%; and 4, >75%. Immunohistochemical staining was performed using antibodies against Kim-1 (1:100), SAM (1:200) and p-S6RP (1:100), as previously described [25]. The semiquantitative analyses of tubular cell damage, Kim-1, SAM and phospho-S6RP in 10 randomly selected tubulointerstitial areas of the renal cortex were evaluated individually per animal as follows: 1, none; 2, minor; 3, moderate; 4, severe; and 5, most severe, in a double-blind manner by two independent observers, as previously described [25]. Anti-mouse monoclonal SAM antibody (MA00201-50) was purchased from Acris Antibodies, Inc. (San Diego, CA, USA). Anti-polyclonal goat Kim-1 antibody (AF3689) was obtained from R&D Systems, Inc. (Minneapolis, MN, USA). Anti-rabbit polyclonal phospho-S6RP (Ser235/236) (#2211) was purchased from Cell Signaling Technology (Danvers, MA, USA).

### Transmission electron microscopy (TEM)

Mitochondrial morphology and fragmented mitochondria in the PTCs were observed using TEM, as previously described [25]. In detail, mitochondrial length was measured in individual tubular cells to determine the percentage of cells showing filamentous mitochondria less than 1% long (>2 μm) [25, 45]. For evaluation of electron microscopic mitochondrial morphology, a total of 18 proximal tubular cells in the control, 22 cells in the diabetes + STD, 18 cells in the diabetes + LPD and 13 cells in the diabetes + LPD + Met group from 3 animals were evaluated.

### Real-time PCR

The isolation of total RNA from the renal cortex, cDNA synthesis and quantitative real-time PCR were performed as previously described [25]. TaqMan probes for *Col3*, Cd68, *Tnf-α*, and *Kim-1* were purchased from Thermo Fisher Scientific (Waltham, MA, USA). The analytical data were adjusted to the level of *18S* mRNA expression as an internal control.

### Western blotting

Western blotting of the renal cortex was performed using antibodies against Gnmt (1:1000), p62 (1:1000), β-actin (1:1000), p-S6RP (1:1000), S6RP (1:1000), Methyl-PP2A, PP2A and LCMT1 as previously described. The anti-rabbit polyclonal p62 antibody (PM045) was obtained from Medical and Biological Laboratories (Nagoya, Japan). Anti-rabbit monoclonal PP2A (#2259), mouse monoclonal LCMT1 (#5691), rabbit polyclonal p-S6RP (Ser235/236) (#2211), and rabbit monoclonal S6RP (#2217) antibodies were obtained from Cell Signaling Technology (Danvers, MA, USA). Anti-mouse monoclonal methylated-PP2A (sc-81603) and mouse monoclonal Gnmt (sc-166834) antibodies were purchased from Santa Cruz Biotechnology (Dallas, TX, USA). Anti-mouse monoclonal β-actin antibodies were obtained from Sigma-Aldrich (St. Louis, MO, USA).

### Measurement of SAM contents in the renal cortex

The contents of SAM in the renal cortex were measured by liquid chromatography/mass spectrometry using LC/MS/MS 8040 (SHIMADZU Co., Kyoto, Japan) at the laboratory of Euglena Co., Ltd. (Tokyo, Japan).

### Autophagic flux in HK-2 cells

HK-2 cells, which are human kidney proximal tubular cells, were obtained from the American Type Culture Collection (ATCC) (Manassas, VA, USA). HK-2 cells were maintained in Keratinocyte-SFM (1×) medium (Life Technologies Green Island NY).

Autophagic flux was determined by analyzing LC3-II turnover by preventing lysosomal degradation using CQ (Sigma-Aldrich, St. Louis, MO, USA). LC3-II expression was evaluated by western blotting using anti-LC3 antibodies (Cell Signaling Technology, Danvers, MA, USA) [37, 46]. LC3-flux was calculated by subtracting the densitometry value of normalized LC3-II in the sample treated with CQ by the value in the sample treated without CQ [37, 46]. LC3-II flux was expressed relative to the respective controls [37, 46]. HK-2 cells were cultured in Hanks’ Balanced Salt Solution (HBSS), amino acid-free solution, with SAM (0.5 mM, 2 mM or 4 mM) or vehicle for 2 hours, and then 50 μM CQ or vehicle was administered for 1 hour before collection of samples.

### Autophagy flux in mice

B6.129-Gnmt^tm1Cwa^/J (glycine N-methyltransferase; targeted mutation 1, Conrad Wagner, Null/Knockout, JAX stock #018066) male and female mice were purchased from Jackson Laboratory (Sacramento, California, USA) [47]. These mice were bred as homozygotes. Male mice with a whole-body KO of *Gnmt* and C57BL/6J mice as wild type littermates were provided free access to food and water. Mice were housed in a temperature- and humidity-controlled room on a 12 h:12 h light-dark cycle. All mice were studied at 8 weeks of age. To assess the autophagic flux by the autophagy-lysosome pathway, mice were injected intraperitoneally with CQ (50 μg/g body weight) and were killed 6 hours after the injection [48]. To study the effects of starvation, mice were completely deprived of food for 48 h (9 a.m. to 9 a.m.) with free access to drinking water. Autophagic flux in the renal cortex was determined by analyzing LC3-II turnover, as described above.

### Effect of SAM on methyl-PP2A and p-S6RP expression in cultured HK-2 cells

SAM (4 mM) was administered to cultured HK-2 cells under HBSS, and samples for western blotting were collected at 0, 15, 30 and 60 min. The expression of methyl-PP2A and p-S6RP was evaluated by western blotting.

### Statistical analysis

The data are expressed as the mean ± standard deviation (SD). A one-way ANOVA or a repeated measures one-way ANOVA followed by Tukey’s multiple comparison test was used to determine the significance of pairwise differences among the four groups. A p value of <0.05 was considered significant.
